# *In situ*-grown hexagonal silicon nanocrystals in silicon carbide-based films

**DOI:** 10.1186/1556-276X-7-634

**Published:** 2012-11-21

**Authors:** Tae-Youb Kim, Chul Huh, Nae-Man Park, Cheol-Jong Choi, Maki Suemitsu

**Affiliations:** 1 Convergence Components and Materials Laboratory, Electronics and Telecommunications Research Institute (ETRI), Daejeon 305-700, Republic of Korea; 2 Department of Semiconductor Science and Technology, Chonbuk National University, Jeonju, 561-756, Republic of Korea; 3 Research Institute of Electrical Communication, Tohoku University, Sendai 980-8577, Japan

**Keywords:** Silicon nanocrystals, *in situ*-formed Si-NCs, Silicon carbide-based films, Hexagonal silicon phase structure

## Abstract

Silicon nanocrystals (Si-NCs) were grown *in situ* in carbide-based film using a plasma-enhanced chemical vapor deposition method. High-resolution transmission electron microscopy indicates that these nanocrystallites were embedded in an amorphous silicon carbide-based matrix. Electron diffraction pattern analyses revealed that the crystallites have a hexagonal-wurtzite silicon phase structure. The peak position of the photoluminescence can be controlled within a wavelength of 500 to 650 nm by adjusting the flow rate of the silane gas. We suggest that this phenomenon is attributed to the quantum confinement effect of hexagonal Si-NCs in silicon carbide-based film with a change in the sizes and emission states of the NCs.

## Background

Silicon-related low-dimensional structures such as Si nanocrystals (Si-NCs) have shown great potential in the development of next-generation devices. When Si-NCs are made smaller than the free-exciton Bohr radius of bulk Si, they behave as quantum dots [1-3] with various energy states that can be tuned using carrier confinement in all three dimensions. These quantum properties of Si-NCs have the greatest impact when they are embedded in a wide-gap dielectric matrix, the structure of which is quite intriguing in the field of Si optoelectronics and third-generation photovoltaics [4,5].

Among such wide-gap dielectric matrices for optoelectronic and photovoltaic devices are Si carbide-based films. These films are considered to have one of the most promising top (window) layers due to a high transparency to photons absorbed by an underneath layer of Si-based junctions as well as to the conductive nature of the material. Moreover, when Si carbide-based film includes Si-NCs, the combination will certainly have further advantages. One such advantage is a lower barrier height caused by a lower bandgap of Si carbide (approximately 2.5 eV) compared to Si oxide (approximately 9 eV) and Si nitride (approximately 5.3 eV), which brings about an increased tunneling probability between Si-NCs [5-7]. Other advantages include the easy formation of minibands between Si-NCs and a higher Bloch carrier mobility [3,8].

To fabricate Si-NCs in Si carbide-based dielectric matrix structures, high-temperature (*T* > 1,100°C) post-deposition annealing of a Si-containing amorphous dielectric film (i.e., Si oxide, Si nitride, and Si carbide) has been commonly used. However, this high-temperature process may pose a serious challenge in the development of a device fabrication process [9]. In this regard, we have developed a method for the *in situ* fabrication of Si-NCs during the deposition of Si nitride-based matrices [10-12]. However, no attempt has yet been made to create a Si carbide-based matrix, which forms the motivation of this study.

Another interesting subject related to the formation of Si-NCs is the control of Si polytypes [13-15]. It is well known that Si crystallizes into a cubic-diamond structure under normal growth and treatment conditions. However, Si is also known to have several polytypes that are stable only at high pressure. Among these Si polytypes, hexagonal-wurtzite Si is important as it sometimes appears in stressed amorphous Si and has enhanced non-linear optical properties for novel optoelectronic applications [13-15].

In this study, we demonstrate the *in situ* formation of hexagonal Si-NCs during the preparation of a silicon carbide-based matrix film at 250°C. The optical gaps of the Si-NCs have been characterized using photoluminescence (PL). Finally, we discuss the size effect of hexagonal Si-NC on its quantum confinement of carriers.

## Methods

Plasma-enhanced chemical vapor deposition (PECVD) [10,12] with methane (CH_4_, >99.999%) and silane (Ar-diluted SiH_4_, >99.9999%) as the reactant gases has been employed to form a Si carbide-based dielectric film. Oxygen atoms are incorporated from the ambient environment, as we will see later. The total pressure and power of the plasma are 0.5 Torr and 5 W, respectively. The use of this low plasma power and highly diluted source gas is proven to be essential in the *in situ* formation of Si-NCs in amorphous Si and Si nitride-based matrices [10,12]. A Si(100) wafer was employed as the sample substrate, whose temperature during deposition was fixed at 250°C. The CH_4_ flow rate was fixed at 10 sccm, while the SiH_4_ flow rate was varied from 10 to 60 sccm to modulate the growth rate of the film. This variation in the SiH_4_ flow rate controls the size of the Si-NCs. The size and crystallinity of the Si-NCs were characterized through high-resolution transmission electron microscopy (HRTEM) using a Tecnai G2 F20 instrument (FEI Co., Hillsboro, OR, USA) operated at 200 kV. To investigate the energy band of the Si-NCs, we conducted PL measurements at room temperature using a He-Cd (325 nm) laser for the excitation. The chemical composition of the matrix film was investigated using Fourier-transformed infrared spectroscopy (FTIR; IFS66V/S and HYPERION 3000, Bruker, Ettlingen, Germany).

## Results and discussion

**Figure 1:**
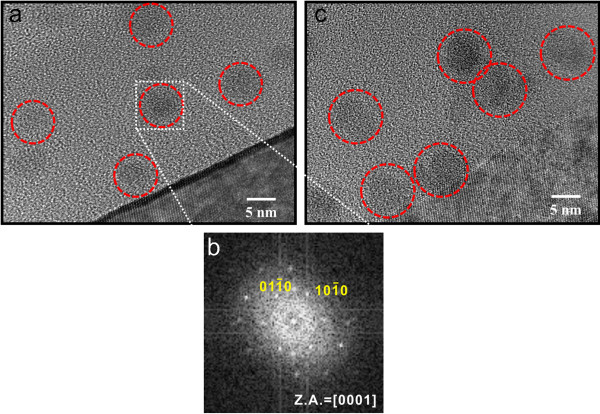
**Cross-sectional HRTEM images of the Si-NCs embedded in a silicon carbide-based film.** (**a**) Approximately 7-nm and (**c**) approximately 9-nm Si-NCs, enclosed by red circles for easy identification. (**b**) Selected area of the electron diffraction pattern from the portion indicated in (a). Presence of (0001)-oriented crystallites of the hexagonal silicon phase is confirmed.

Figure 1a,c shows HRTEM images of the deposited film. In Figure 1a, the sample was grown using the gas flow rates of SiH_4_/CH_4_ = 20:10 sccm, and the average size of the Si-NCs is approximately 7 nm. In Figure 1c, the sample was grown using the gas flow rates of SiH_4_/CH_4_ = 60:10 sccm, and the average size of the Si-NCs is approximately 9 nm. Most of the Si-NCs are in a crystalline state, as evidenced by the lattice fringe shown in the HRTEM image as well as from the spotty pattern shown in the selected area of electron diffraction from one of the crystallites (Figure 1b). The diffraction pattern was determined to be caused by a (0001)-oriented hexagonal Si crystal [14,16]. From the diffraction pattern, the plane separation is determined to be d(10 Ī 0) = 3.31 Å, which is in good agreement with the established value (3.29 Å) for the hexagonal Si phase (JCPDS: Powder Diffraction File #80-0005).

**Figure 2:**
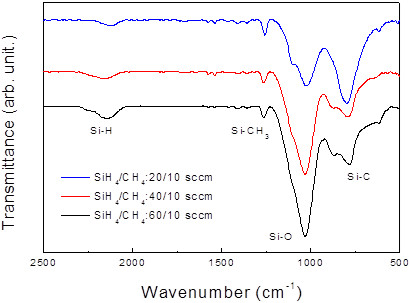
**Fourier transform infrared spectroscopy spectra.** The spectra as a function of SiH_4_ flow rate at a fixed CH_4_ flow rate of 10 sccm. Four absorption peaks were observed, and the peak intensities varied with the SiH_4_ flow rate.

The chemical composition of the dielectric matrix changes with the SiH_4_ flow rate. Figure 2 shows a series of FTIR spectra for three different SiH_4_ flow rates. The intense absorption bands at 780 to 800 cm^−1^ and 1,030 to 1,040 cm^−1^ are assigned to the Si-C and Si-O stretching modes [17,18], while the bands at 1,250 to 1,260 cm^−1^ and 2,120 to 2,150 cm^−1^ are assigned to the Si-CH_3_ and Si-H stretching modes [17-19]. The dominance of the Si-O band, as well as the absence of C=O bonds (approximately 1,700 cm^−1^), indicates that the film is of a Si carbide-based matrix with most of the oxygen atoms bonded to Si atoms. Although we do not exclude the presence of Si-O-C bonding, its absorption peak at 1,125 cm^−1^ is very faint. The incorporation of oxygen atoms may be due either to the low purity of the nitrogen gas used to vent the reactor chamber or to the incorporation of oxygen atoms from the rather high (10^−1^ Pa) base pressure of the PECVD system. The very slow (<2 nm min^−1^) growth condition necessary to realize the *in situ* formation of Si-NCs [10,20,21] accelerates the incorporation of oxygen atoms. However, the Si-O peak intensity increases with the SiH_4_ flow rate. This is best understood in terms of the stronger bonding between Si and O atoms, which can be observed in these very slow growth conditions.

The growth mechanism of Si-NCs in a Si carbide-based film may be identical to the *in situ* formation of Si-NCs in amorphous Si [20] and Si nitride-based matrix films [10,12]. Namely, the key condition is the low rate of film growth, which can be acquired by low plasma power as well as by a high dilution of the source gas. Under optimum conditions, the Si nanoparticles are dispersedly formed in the matrix films during the film growth [12,20,21]. The Si nanoparticles formed are then transformed into nanocrystalline Si by hydrogen radical diffusion from the amorphous Si nanoparticles [10,12]. It is then likely that the structure of the Si-NCs is influenced by their surrounding matrix at the moment of crystallization. Namely, one possible mechanism for the formation of hexagonal-wurtzitic Si-NCs is the surrounding oxygen and carbon atoms (network) inducing sufficient stresses on the nuclei [13,15].

**Figure 3:**
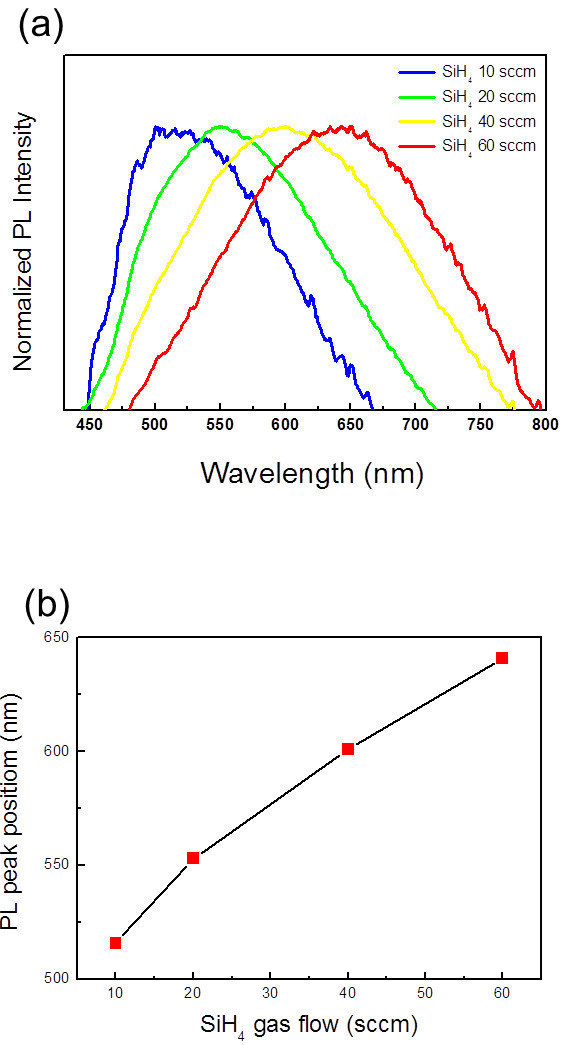
**Photoluminescence spectra.** (**a**) The spectra of samples grown by changing the flow rate of SiH_4_ gas at a fixed CH_4_ gas flow rate of 10 sccm and (**b**) PL peak positions as functions of the flow rates of SiH_4_ gas.

**Figure 4:**
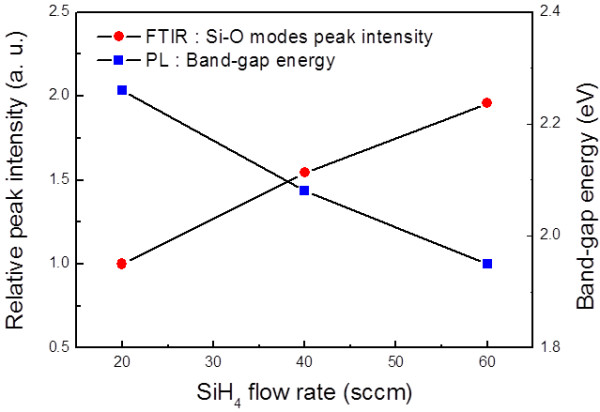
**Relative FTIR absorption peak intensities of Si-O stretching mode and PL bandgap energy.** As a function of the SiH_4_ flow rate.

One of the most intriguing electronic properties in semiconducting nanostructures is the tunability of the bandgap based on their sizes. Figure 3a shows a series of PL spectra obtained for materials grown using SiH_4_ flow rates of 10, 20, 40, and 60 sccm with a fixed CH_4_ flow rate of 10 sccm. With an increase in the silane flow rate, the peak position shows a redshift (see also Figure 3b). The possible origin of the PL emission is three-fold in our samples: (1) recombination at Si dangling bonds of defects, (2) recombination in the surrounding amorphous SiC-based matrix, and (3) recombination between quantized conduction and valence band states within the Si-NCs. Among the three, the first possible origin is denied because Si dangling bonds cannot have the observed variation in the PL energy [22]. The second possible origin is also denied because the optical bandgap of a-SiC-based film, and hence the PL energy, should increase with the incorporation of carbon or oxygen into the matrix in this model [23,24]. As we will see in Figure 4, however, the PL energy decreases with the increase of the oxygen content in the matrix.

We suggest the third possibility, the quantum confinement effect, being the most likely origin of the PL emission. As we have seen in Figure 1a,c, the PL redshift with increasing SiH_4_ flow rate (Figure 3b) is accompanied by enlargement of the Si-NC size. This tendency between the PL energy versus Si-NC size *d* is consistent with the reported value based on the quantum confinement effect of *A + B/d^*2 with *A* = 1.475 eV and *B* = 38.5 (*A* is the bulk bandgap of the hexagonal crystal silicon, and *B* is the confinement parameter) [10,13,21], although further HRTEM observations are needed to determine the intermediate *d* values required for a more detailed analysis. Still, this *A* value is in reasonable agreement with the theoretical value of the direct-gap energy of approximately 1.48 eV by Joannopoulos and Cohen [25] and the experimental value (PL gap energy) of approximately 1.45 [13] both for hexagonal-wurtzite Si crystal.

The FTIR results of the Si-O peak intensity and PL spectra provide further support for the dominance of the quantum confinement effect. Figure 4 shows the relative FTIR absorption peak intensities of the Si-O stretching mode and the PL bandgap energy as a function of the SiH_4_ flow rate. As the SiH_4_ flow increases, the PL energy decreases while the Si-O mode intensity increases. Increasing the Si-O peak intensity means the increase of the oxygen content in the matrix, and it means the increase of the optical bandgap*.* The lowering of the PL energy, however, despite the increase of the oxygen content in the matrix, clearly indicates the dominance of the quantum confinement effect in determining the PL energy of the Si-NCs. We can therefore suggest that the hexagonal Si-NCs may have a tunable bandgap energy based on their size through the quantum confinement effect.

## Conclusions

We investigated the *in situ* formation of Si-NCs during the growth of the surrounding Si carbide-based film through PECVD using methane (CH_4_) and silane (SiH_4_). No post-annealing process is necessary. The HRTEM measurements and PL results indicate the formation of Si-NCs with a hexagonal structure and good tunability of the optical bandgap under the growth conditions. The blueshift in the PL peak with a decreasing SiH_4_ flow rate can be attributed to the three-dimensional quantum confinement effect in the hexagonal Si-NCs. These results demonstrate the viable potential of this method for the fabrication of silicon-based third-generation photovoltaics.

## Competing interests

The authors declare that they have no competing interests.

## Authors' contributions

TYK and MS designed the study and carried out the experiments. TYK, CH, NMP, and CJC performed the treatment of experimental data and calculations. TYK, CH, NMP, and MS took part in the discussions of the results and prepared the manuscript initially. All authors read and approved the final manuscript.
